# Topological Data Analysis-Driven fNIRS Signal Processing for Alzheimer’s Disease Stage Identification

**DOI:** 10.3390/s26165221

**Published:** 2026-08-18

**Authors:** Siyuan Liu, Hangcheng Wu, Cheng Sun, Yuanbin Qiu, Haoliang Wu, Yucong Wei, Yang Lv, Zheng Yang

**Affiliations:** 1 Pittsburgh Institute, Sichuan University, Chengdu 610207, China; liusiyuan0817@126.com (S.L.); 2023141520244@stu.scu.edu.cn (H.W.); 2023141520230@stu.scu.edu.cn (C.S.); 2024141520345@stu.scu.edu.cn (Y.Q.); yucong.wei@scupi.cn (Y.W.); 2 West China School of Clinical Medicine, Sichuan University, Chengdu 610041, China; 2022151620321@stu.scu.edu.cn; 3 Department of Geriatrics, The First Affiliated Hospital of Chongqing Medical University, Chongqing 400016, China; yanglyu@hospital.cqmu.edu.cn

**Keywords:** topological data analysis (TDA), persistent homology, persistence images, fNIRS, feature extraction, Alzheimer’s disease, granger causality, brain networks

## Abstract

This paper proposes a novel Topological Data Analysis (TDA) pipeline to extract robust structural features from functional near-infrared spectroscopy (fNIRS) signals for the classification of Alzheimer’s Disease (AD) stages. Alzheimer’s disease is increasingly understood as a disconnection syndrome, where the disruption of functional brain networks precedes gross anatomical atrophy. However, traditional graph-theoretic approaches rely on arbitrary connectivity thresholds, which can obscure critical multi-scale topological information, and are sensitive to noise. To address this, our framework leverages Persistent Homology (PH) to analyse the topological evolution of brain networks across a continuous range of scales. By modeling 48-channel hemoglobin concentration time-series as high-dimensional point clouds via Granger causality metrics, we construct filtration sequences of Vietoris–Rips complexes. The resulting topological invariants, including 0—dimensional connected components, 1—dimensional loops, and 2—dimensional voids, are first examined through Persistence Diagrams. For classification, significant H0 and H1 features are converted into Persistence Images using Gaussian kernel smoothing, while H2 features are retained for qualitative topological interpretation. This transformation enables the integration of complex topological features into standard machine learning workflows. Our experimental results were evaluated on a subject-level held-out test set consisting only of original, non-augmented recordings. Data augmentation was applied only to the training set to alleviate class imbalance. The proposed topology-driven feature extraction method achieved 86% accuracy in multi-class diagnosis (NC vs. MCI vs. AD). This study validates the efficacy of TDA as a sophisticated signal processing tool for revealing intrinsic neurodegenerative patterns in hemodynamic data, offering an exploratory methodological proof-of-concept for AD stage classification.

## 1. Introduction

Alzheimer’s disease (AD) is a progressive neurodegenerative disorder and the leading cause of dementia worldwide. As the global population ages, the prevalence of AD is projected to rise dramatically, posing a significant burden on healthcare systems [1]. The disease pathology is characterized by the accumulation of β-amyloid plaques and neurofibrillary tangles, which lead to synaptic loss and neuronal death [2]. Clinically, the disease progresses through a continuum: from Normal Cognition (NC) to Mild Cognitive Impairment (MCI)—a prodromal stage where intervention is most effective—and finally to AD dementia [3,4]. Consequently, developing accurate, non-invasive, and cost-effective tools for early-stage classification, particularly distinguishing MCI from NC, is a critical research priority.

While neuroimaging modalities such as Magnetic Resonance Imaging (MRI) and Positron Emission Tomography (PET) are the gold standards for diagnosis [5,6], they are often expensive, immobile, and contraindicated for patients with metallic implants or claustrophobia. Functional Near-Infrared Spectroscopy (fNIRS) has emerged as a promising alternative. By monitoring hemodynamic responses (oxy-hemoglobin HbO and deoxy-hemoglobin HbR) in the cortex, fNIRS provides a measure of neurovascular coupling similar to fMRI but with higher temporal resolution, lower cost, and greater portability [7,8,9].

However, analyzing fNIRS data for AD classification presents unique signal processing challenges. The “disconnection hypothesis” suggests that cognitive decline in AD arises from the disruption of functional integration between distributed brain regions rather than damage to a single area. Traditional network analysis typically involves constructing a functional connectivity graph by thresholding a correlation matrix. This approach suffers from two major limitations:

(1) Threshold Arbitrariness: Choosing a threshold to binarize the network is subjective. A high threshold may fracture the network, losing weak but biologically significant connections, while a low threshold may introduce noise.
(2) Scale Sensitivity: Brain networks exhibit multiscale organization. Fixed-scale graph metrics (e.g., path length, clustering coefficient) fail to capture the hierarchical topology of neural degeneration.

To overcome these limitations, we propose a Topological Data Analysis (TDA) framework based on Persistent Homology (PH). TDA treats data as a shape and analyzes its connectivity across all possible thresholds simultaneously (a process called filtration) [10,11]. This renders the analysis robust to noise and independent of arbitrary parameter choices. While TDA has been applied to MRI and PET data [12,13], its application to fNIRS-derived effective connectivity networks remains underexplored.

In this work, we develop a comprehensive pipeline that:

- Transforms raw fNIRS time-series into Effective Connectivity networks using Granger Causality, capturing the directionality of information flow.
- Extracts topological invariants (Betti numbers) via Persistent Homology, identifying stable structures (clusters, loops and higher-order voids) that persist across scales.
- Vectorizes these abstract topological features into Persistence Images (PIs), creating a stable, fixed-dimensional input for machine learning classifiers.

**Figure 1:**
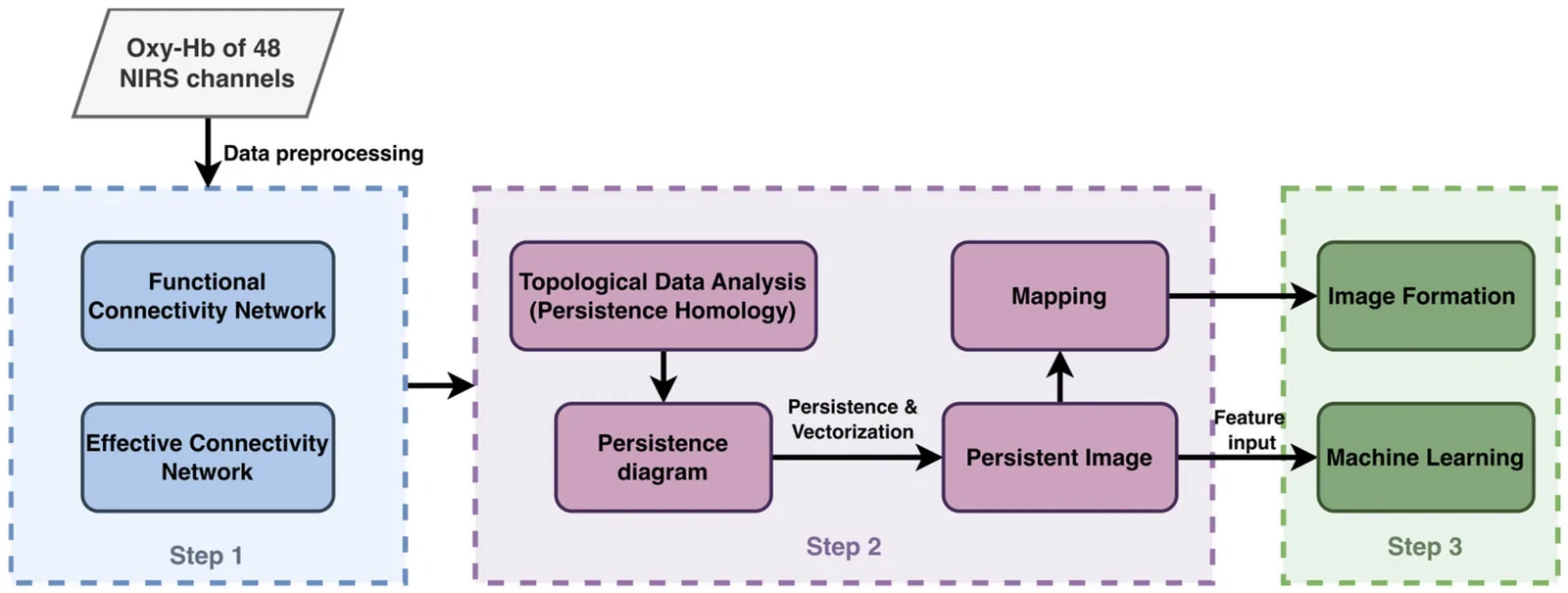
Proposed TDA-based data analysis pipeline: From fNIRS signal acquisition to topological feature vectorization and classification. The pipeline emphasizes the transformation from temporal signal space to topological feature space.

We demonstrate that this topology-aware machine learning approach significantly outperforms baseline methods, providing a robust framework for automated AD staging. The workflow is illustrated in Figure 1.

## 2. Literature Review

### 2.1. Machine Learning in AD Diagnosis

The application of machine learning to neuroimaging has been extensive. Early approaches relied on hand-crafted features fed into Support Vector Machines (SVMs) [14]. More recently, deep learning, particularly Convolutional Neural Networks (CNNs), has achieved state-of-the-art results on structural MRI [15,16,17]. For instance, Suk et al. [18] utilized Deep Boltzmann Machines for multimodal fusion, while Liu et al. [19] explored manifold learning. Despite these successes, deep learning models often function as “black boxes,” lacking interpretability regarding the underlying network failure mechanisms. Furthermore, their performance on early-stage MCI detection often plateaus between 60% and 80% [6,20], suggesting that standard morphological or intensity-based features may miss subtle global connectivity changes.

### 2.2. fNIRS in Cognitive Neuroscience

fNIRS has gained traction for studying neurodegenerative diseases due to its sensitivity to neurovascular coupling changes. Research indicates that AD patients exhibit reduced hemodynamic activation in the prefrontal and parietal cortices during cognitive tasks [8]. However, most fNIRS studies rely on localized activation analysis or simple functional connectivity metrics (Pearson correlation). These methods often overlook the directional and causal relationships between brain regions. Effective connectivity, assessed via Granger Causality, provides a more rigorous description of neural communication [21], yet its combination with advanced topology remains rare in fNIRS literature.

### 2.3. Topological Data Analysis (TDA) in Medicine

TDA has recently emerged as a powerful tool for quantifying the “shape” of complex biological data. In the context of functional neuroimaging, TDA has been successfully applied to map dynamic brain states. For instance, Saggar et al. [22] demonstrated that TDA could reveal the dynamical organization of the brain, capturing transitions in functional connectivity that traditional metrics overlook. Similarly, Anderson et al. [23] utilized persistent homology to identify behaviorally relevant topological features in functional networks. The core advantage of TDA is its ability to separate true signal from noise based on the “persistence” of topological features. Unlike traditional graph theory, which relies on arbitrary thresholding and captures only local properties, TDA captures global properties (holes, voids, and connectivity) that are invariant under continuous deformation.

## 3. Materials & Methodology

### 3.1. Participants and Experimental Protocol

Data were obtained from a retrospective, de-identified fNIRS dataset consisting of 284 participants, categorized into three groups: Normal Cognition (NC), Mild Cognitive Impairment (MCI), and Alzheimer’s Disease (AD). Written informed consent had been obtained from all participants at the time of the original data collection. The present study involved only secondary analysis of de-identified recordings and did not involve direct participant contact or additional data collection.

Participants were categorized into NC, MCI, and AD groups according to clinical diagnosis based on standard criteria. The NC group included cognitively normal participants without clinically significant cognitive complaints. The MCI group included participants with measurable cognitive decline that did not substantially interfere with daily living activities. The AD group included participants with clinically diagnosed Alzheimer’s disease dementia.

The original dataset consisted of 284 independent fNIRS recordings, including 134 AD recordings, 84 MCI recordings, and 66 NC recordings. Before data augmentation, 130 original recordings were reserved as an independent held-out test set, including 65 AD, 37 MCI, and 28 NC recordings. The remaining 154 recordings, including 69 AD, 47 MCI, and 38 NC recordings, were used for model training.

The inclusion criteria were as follows: availability of valid fNIRS recordings and complete diagnostic label for NC, MCI, or AD. Participants were excluded if recordings were of insufficient quality after preprocessing.

Note that detailed age, sex, education, and cognitive assessment scores (MMSE/MoCA/CDR) were not available in this de-identified dataset and are acknowledged as limitations.

#### 3.1.1. Dataset Balancing via Augmentation

The original dataset was imbalanced, with fewer MCI and NC cases compared with AD. To mitigate class imbalance, data augmentation was applied only to the training data after subject-level splitting. Specifically, 130 original fNIRS recordings were first reserved as an independent held-out test set. The remaining 154 original recordings were used for model training. Gaussian noise was then injected only into the raw fNIRS time series of the training recordings, increasing the number of training samples from 154 to 520.

The augmented signal was defined as:

(1) $$X_{\mathrm{aug}}\left(t\right)=X_{\mathrm{raw}}\left(t\right)+N\left(0,\sigma_{\mathrm{noise}}^{2}\right)$$

where $\sigma_{noise}$ was set to 0.05 times the standard deviation of the corresponding original signal. The perturbation magnitude was deliberately kept small to introduce signal-level variability while approximately preserving the temporal structure of the fNIRS recordings.

No augmented sample was generated from or included in the independent held-out test set. In addition, during hyperparameter tuning with 5-fold cross-validation on the training set, augmentation was performed only within the training fold of each split; the corresponding validation fold remained composed of original, non-augmented recordings. Therefore, synthetic variants of the same original recording could not appear simultaneously in training and validation/test subsets. The reported held-out test performance in Section 4 was evaluated only on original, non-augmented recordings.

#### 3.1.2. Hyperparameter Optimization

Hyperparameter tuning was conducted using 5-fold cross-validation on the training set, while final model evaluation was performed on the independent held-out test set. Data splitting was performed at the subject level, with stratification to maintain class balance in each fold. The random seed was fixed (seed = 77) to ensure reproducibility of the training and validation splits.

Within each fold, data augmentation was applied only to the training portion, while the validation fold contained only original, non-augmented recordings. Model hyperparameters were selected based on the mean macro-F1 score across the validation folds.

The search space included:

- Random Forest: Number of trees $\in \left[50,100,200\right]$, Max depth $\in \left[None,10,20\right]$.
- XGBoost: Learning rate $\in \left[0.01,0.1,0.2\right]$, Gamma $\in \left[0,0.1,0.2\right]$.
- PI Resolution: For persistence image resolution, grid sizes from 1 × 1 to 15 × 15 were evaluated during hyperparameter selection. The 9 × 9 grid achieved the best performance and was therefore used in the final model.

#### 3.1.3. Evaluation Metrics

We evaluated the classification performance using standard metrics derived from the confusion matrix elements (True Positives TP, False Positives FP, False Negatives FN):

- Accuracy: The ratio of correctly predicted observations to the total observations.
- Precision: The ratio of correctly predicted positive observations to the total predicted positives: $\mathrm{Precision}=\frac{TP}{TP+FP}$
- Recall (Sensitivity): The ratio of correctly predicted positive observations to all observations in actual class: $\mathrm{Recall}=\frac{TP}{TP+FN}$
- F1-Score: The weighted average of Precision and Recall:

(2) $$F1=2\cdot \frac{\mathrm{Precision}\cdot \mathrm{Recall}}{\mathrm{Precision}+\mathrm{Recall}}$$

Given the multi-class nature of the problem, we report the macro-average for these metrics.

### 3.2. fNIRS Data Acquisition and Detailed Preprocessing

Hemodynamic signals were recorded using a multi-channel fNIRS system (NirScan, Danyang Huichuang Medical Equipment Co., Ltd., Zhenjiang, China). The system utilized two wavelengths of near-infrared light, 760 nm and 850 nm, with a sampling rate of 11 Hz. The probe geometry consisted of 15 sources and 16 detectors, arranged to create 48 measurement channels with a source-detector separation of 3 cm. The optodes were positioned according to the international 10–20 system, covering the bilateral prefrontal cortex (PFC) and temporal cortices. Specifically, the probe array was centered on the Fpz channel of the international 10/20 system, with the lowest probes aligned along the Fp1–Fp2 line. The PFC is heavily implicated in executive dysfunction in AD.

The raw light intensity data contains significant physiological noise (e.g., Mayer waves, respiration, heart rate) and instrumental noise. Our preprocessing pipeline was rigorously designed as follows:

(1) Optical Density Conversion

The raw intensity *I*(*t*) was converted to optical density change relative to a baseline reference *I*_0_:

(3) $$\Delta OD\left(t\right)=-\log_{10}\left(\frac{I\left(t\right)}{I_{0}}\right)$$

The baseline reference *I*_0_ was strictly defined as the mean intensity during the 10-s resting-state period immediately preceding the cognitive task.

(2) Motion Artifact Correction (Moving Standard Deviation):

Motion artifacts are often characterized by sudden spikes. We employed a Moving Standard Deviation (MSD) method with a sliding window size set precisely to 1 s (11 frames at 11 Hz). An artifact threshold was defined as 3 standard deviations above the local mean. If σ(t) > σ_threshold_, the segment was identified as an artifact. These segments were corrected using Cubic Spline Interpolation over the detected artifact window plus a 0.5-s buffer on both ends to preserve signal continuity. Additionally, a strict channel rejection criterion was applied: any channel with a Signal-to-Noise Ratio (SNR) <2 or flatlining for >5 s was excluded from subsequent analysis.

(3) Band-pass Filtering:

To isolate the task-evoked hemodynamic response, we applied a Finite Impulse Response (FIR) band-pass filter with a passband of 0.01–0.20 Hz. This range effectively removes:

High-frequency cardiac noise (∼1.0–1.2 Hz).

Respiratory noise (∼0.3 Hz).

Very low-frequency drift (<0.01 Hz).

(4) Modified Beer–Lambert Law (MBLL):

The filtered optical density changes were converted into concentration changes of oxygenated hemoglobin ($\Delta \left[HbO\right]$) and deoxygenated hemoglobin ($\Delta \left[HbR\right]$). A fixed differential path length factor (*DPF*) of 6.0 was applied across all channels. The MBLL accounts for the scattering of light in biological tissue:

(4) $$\left[\begin{array}{c} \Delta [HbO](t)\\ \Delta [HbR](t) \end{array}\right]=\frac{1}{d\cdot DPF}{\left[\begin{array}{c} \epsilon_{HbO}^{\lambda_{1}}\;\;\;\;\epsilon_{HbR}^{\lambda_{1}}\\ \epsilon_{HbO}^{\lambda_{2}}\;\;\;\;\epsilon_{HbR}^{\lambda_{2}} \end{array}\right]}^{-1}\left[\begin{array}{c} \Delta OD^{\lambda_{1}}(t)\\ \Delta OD^{\lambda_{2}}(t) \end{array}\right]$$

$\Delta \left[HbO\right]$ was prioritized for subsequent analysis due to its higher signal-to-noise ratio (SNR) compared to $\Delta \left[HbR\right]$. Ultimately, this preprocessing pipeline yielded a 48 × 1600 fNIRS time-series dataset for each participant.

Here, *d* = 3.0 cm is the inter-optode distance. DPF=6.0 is the differential path length factor, representing the ratio of the actual photon path length to the inter-optode distance due to scattering. ϵ terms are the wavelength-specific molar extinction coefficients.

### 3.3. Metric Space Construction: Connectivity Networks

TDA requires the data to be represented as a metric space or a weighted graph. We utilized Granger Causality to define the “effective distance” between brain regions.

We prioritized $\Delta \left[HbO\right]$ for subsequent topological analysis, as previous studies suggest it provides a more robust measure of local cerebral blood flow changes in cognitive tasks.

**Figure 2:**
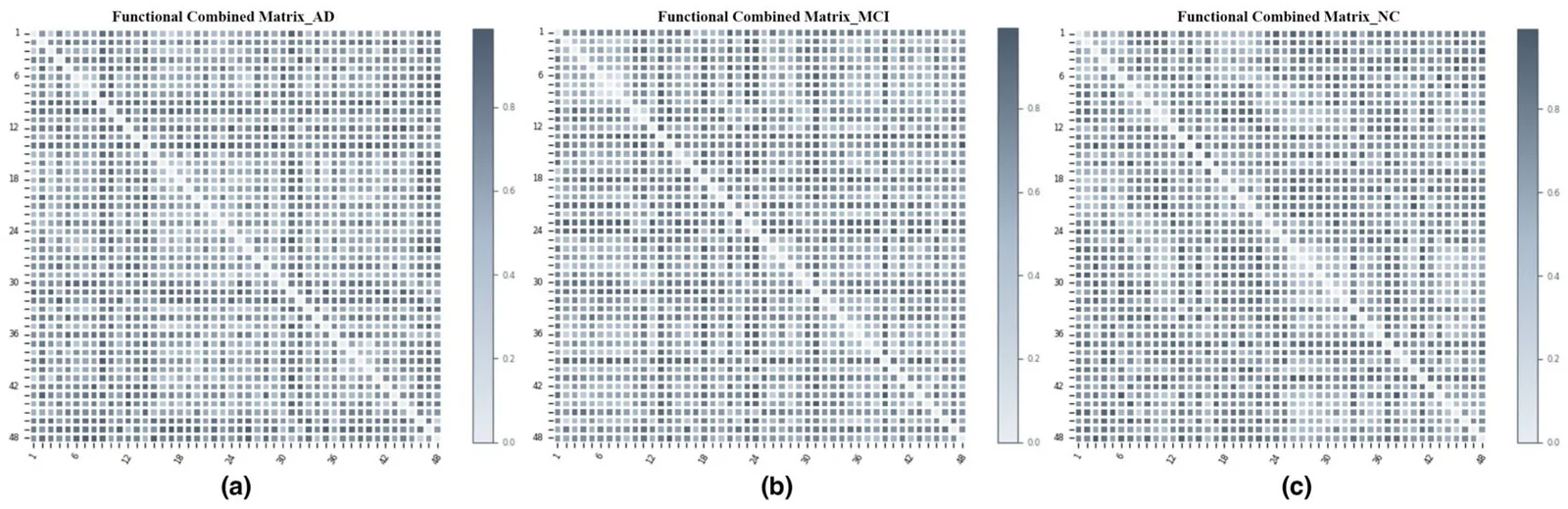
Functional connectivity network based on Pearson correlation, highlighting undirected statistical dependencies between brain regions. Subpicture (**a**–**c**) show different results of AD, MCI and NC.

**Figure 3:**
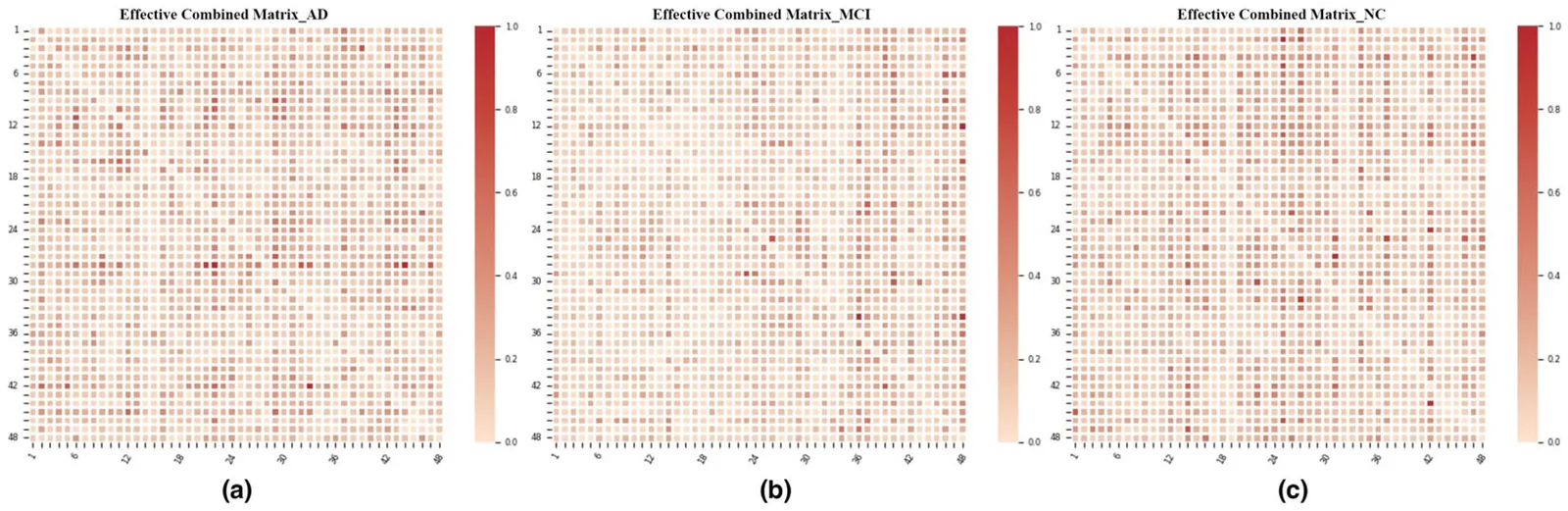
Effective connectivity network constructed using Granger Causality Analysis (GCA), illustrating the directional information flow and causal influence, which is crucial for analysing the “disconnection syndrome” in Alzheimer’s Disease. Subpictures (**a**–**c**) show different results of AD, MCI and NC.

While functional connectivity (Pearson correlation) captures statistical dependence, it is inherently undirected (as illustrated in Figure 2, which depicts a standard functional connectivity network). However, the “disconnection syndrome” in AD implies a disruption in the directional information flow between brain regions. Granger Causality Analysis (GCA) allows us to infer this directional influence, enabling the construction of an effective connectivity network that maps the asymmetric flow of information (Figure 3).

We modeled the time series using multivariate autoregressive (MVAR) models. For two time series, $X_{1}\left(t\right)$ and $X_{2}\left(t\right)$, the causality from $X_{2}$ to $X_{1}$ is tested by comparing the prediction error of two models.

The Unrestricted Model (incorporating history of both variables):

(5) $$X_{1}\left(t\right)=\sum_{j=1}^{p}A_{11,j}X_{1}\left(t-j\right)+\sum_{j=1}^{p}A_{12,j}X_{2}\left(t-j\right)+E_{1U}\left(t\right)$$

The Restricted Model (incorporating history of $X_{1}$ only):

(6) $$X_{1}\left(t\right)=\sum_{j=1}^{p}A_{11,j}^{\prime }X_{1}\left(t-j\right)+E_{1R}\left(t\right)$$

Here, *p* is the model order, selected via the Akaike Information Criterion (AIC) to balance model complexity and goodness of fit. In this study, we determined the optimal order to be *p* = 15 (approximately 1.4 s), sufficient to capture hemodynamic lags.

The magnitude of Granger causality *F*_2→1_ is quantified as:

(7) $$F_{2\to 1}=\ln\left(\frac{var\left(E_{1R}\right)}{var\left(E_{1U}\right)}\right)$$

where $var\left(\cdot \right)$ denotes the variance of the residual errors. If $X_{2}$ contains unique information about the future of $X_{1}$, the unrestricted error $E_{1U}$ will be significantly smaller than $E_{1R}$, yielding a positive F value.

This computation results in a 48×48 asymmetric adjacency matrix for each subject. For TDA input, which typically operates on metric spaces, we symmetrized this matrix to define edge weights $W_{ij}$

(8) $$W_{ij}=\frac{F_{i\to j}+F_{j\to i}}{2}$$

Symmetrization ensures compatibility with persistent homology computations while providing robust estimation of global topological features. Although this step removes fine-grained directional information, prior work has demonstrated that undirected topological features can effectively capture and discriminate brain state differences. For example, TDA approaches using persistent homology have successfully revealed behaviorally relevant brain dynamics and global connectivity patterns in fMRI studies of cognitive tasks and individual differences, even when derived from undirected functional connectivity networks [22,23].

Although this step reduces fine-grained directional information, it is standard practice in current TDA-based neuroimaging pipelines to ensure mathematical consistency for persistent homology computations. We prioritize global topological stability, while acknowledging that future implementations using directed filtration methods will aim to retain the full causal asymmetry derived from Granger Causality.

To convert these weights (strength) into distances (dissimilarity), we applied the inversion: $D_{ij}=1/W_{ij}$ (normalized).

### 3.4. Topological Data Analysis: Persistent Homology

The core of our proposed method is the extraction of robust topological features. While traditional graph-theoretic metrics (e.g., degree, efficiency, small-worldness) provide valuable insights, they rely heavily on a fixed threshold to binarize the connectivity matrix. This thresholding process is often arbitrary and can lead to the loss of critical information: a high threshold may fracture weak but biologically significant connections, while a low threshold introduces noise. To overcome this limitation, we employ Persistent Homology (PH), a method rooted in algebraic topology. PH tracks the evolution of topological features across a continuous range of scales (filtration), thereby providing a multi-scale summary of the brain network’s intrinsic structure that is invariant to continuous deformations and robust to noise.

#### 3.4.1. Mathematical Foundations: From Simplices to Homology Groups

We model the functional brain network as a discrete combinatorial structure known as a Simplicial Complex.

Simplex means given a set of vertices V (in our case, the 48 fNIRS channels); a k-simplex $\sigma_{k}$ is a convex hull of k+1 affinely independent vertices $\left\{v_{0},v_{1},\ldots ,v_{k}\right\}$. Specifically, 0-simplex is a vertex (brain region), 1-simplex is an edge (functional connection), and 2-simplex is a triangle (functional triplet/clique).

A simplicial complex K is a collection of simplices such that: if σ∈K, then every face of σ is also in K. The intersection of any two simplices in K is a face of both.

To rigorously quantify the “holes” or voids in the complex, we introduce the concept of Homology Groups over a field $F_{2}$ (typically $Z_{2}$ for computational efficiency). Let $C_{k}$ be the vector space generated by the k-simplices of K. We define the Boundary Operator $\partial_{k}:C_{k}\to C_{k-1}$ as a linear map that sends a simplex to the formal sum of its $\left(k-1\right)$-dimensional faces. A fundamental property of the boundary operator is $\partial_{k-1}\circ \partial_{k}=0$, which implies that the boundary of a boundary is empty.

Based on this, we define two subspaces:

Cycle Group ($Z_{k}$): The kernel of the boundary operator, $Z_{k}=\ker\partial_{k}=\{c\in C_{k}|\partial_{k}c=0\}$. Elements of $Z_{k}$ represent cycles (loops, voids) that have no boundary.

Boundary Group ($B_{k}$): The image of the boundary operator, $B_{k}=\mathrm{im}\partial_{k+1}=\{c\in C_{k}|\exists d\in C_{k+1},\partial_{k+1}d=c\}$. Elements of $B_{k}$ are cycles that are “filled in” by higher-dimensional simplices.

The k-th Homology Group $H_{k}\left(K\right)$ is defined as the quotient group:

(9) $$H_{k}\left(K\right)=\frac{Z_{k}}{B_{k}}$$

This algebraic structure effectively filters out “trivial” cycles (those that are boundaries of solid shapes) and retains “non-trivial” holes. The rank of the homology group is the Betti number $\beta_{k}=\dim\left(H_{k}\right)$, which serves as a topological invariant.

By analyzing the Betti numbers across this filtration process, we can derive meaningful neurobiological insights:

$\beta_{0}$: Number of connected components. In the context of AD, a higher $\beta_{0}$ at late filtration stages indicates network fragmentation and a lack of global integration.

$\beta_{1}$: Number of 1-dimensional holes (loops). Physically, these represent redundant pathways for information flow. A reduction in $\beta_{1}$ suggests a loss of cognitive reserve and network resilience.

$\beta_{2}$: Number of 2-dimensional voids or cavities. Physically, these represent higher-order functional cliques and complex 3D integration pathways among multiple brain regions. A reduction in $\beta_{2}$ indicates a breakdown of higher-order cognitive coordination and a disrupted global functional architecture.

#### 3.4.2. The Vietoris–Rips Filtration

Since the brain network is a weighted graph derived from Granger Causality, it naturally possesses a metric structure rather than a fixed binary structure. To analyze the topology across all possible thresholds, we construct a Filtration.

Let *V* be the set of channels and D be the distance matrix, where $D_{ij}$ represents the dissimilarity (inverse of causal strength) between channel i and j. For a scale parameter ϵ≥0, the Vietoris–Rips Complex $Rips_{\epsilon }\left(V\right)$ is defined as:

(10) $$\sigma \in {Rips}_{\epsilon }\left(V\right)⟺\forall u,v\in \sigma ,D\left(u,v\right)\leq \epsilon$$

This definition implies that a simplex is formed if and only if the pairwise distances between all its vertices are at most ϵ. As ϵ increases from 0 to $\epsilon_{max}$, we generate a nested sequence of simplicial complexes, known as the filtration:

(11) $$\emptyset \subseteq K_{\epsilon_{0}}\subseteq K_{\epsilon_{1}}\subseteq \cdots \subseteq K_{\epsilon_{n}}=K_{\mathrm{final}}$$

**Figure 4:**
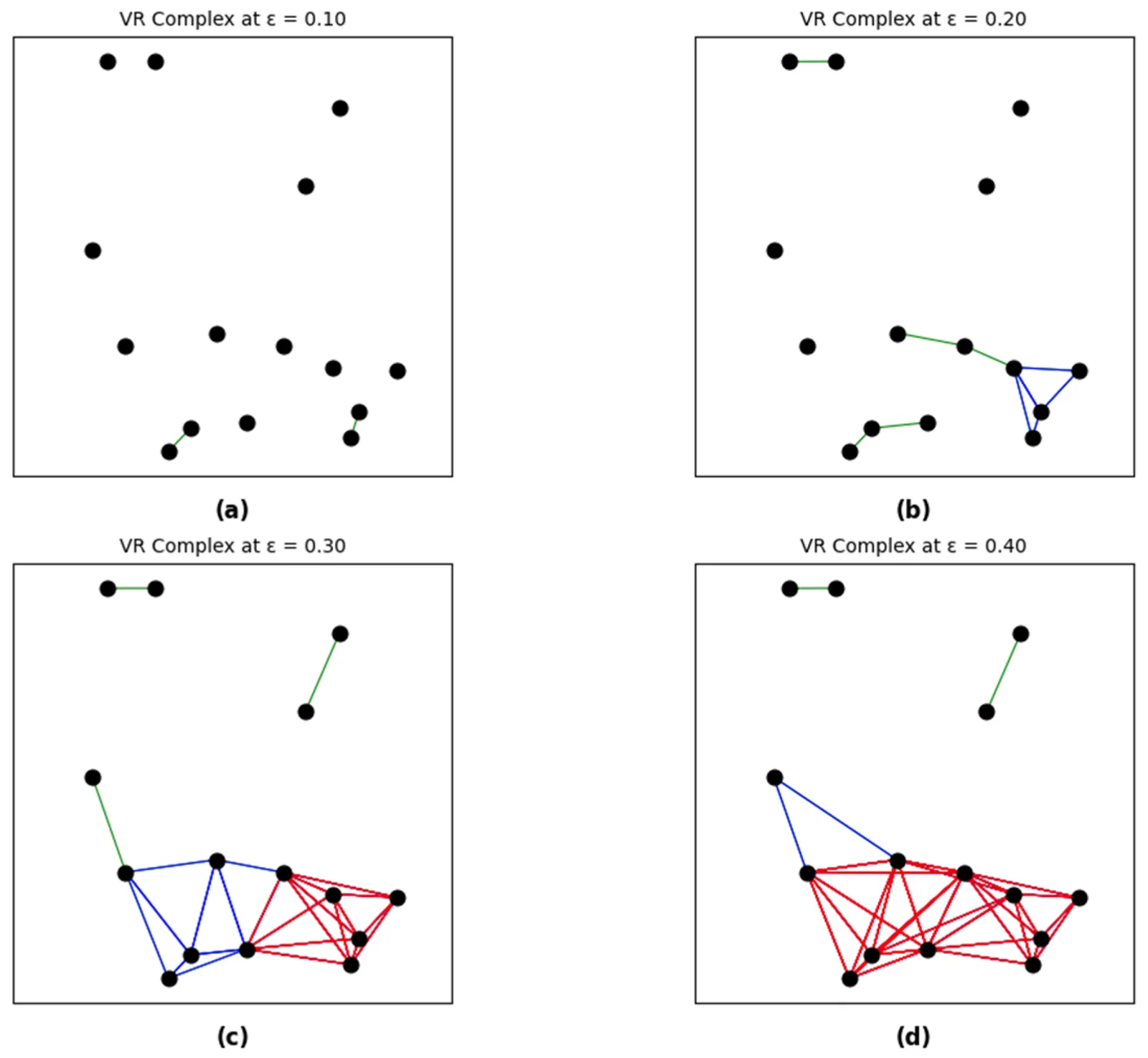
Subfigures (**a**–**d**) show the schematic of the Rips Filtration process. As the scale parameter ϵ increases, points are connected to form simplicial complexes. We track the ‘birth’ (ϵ_birth_) and ‘death’ (ϵ_death_) of topological features.

As illustrated in Figure 4, this filtration simulates the dynamic evolution of the brain’s functional architecture by systematically varying the connection threshold:

(1) At ϵ=0 (or low ϵ, e.g., ϵ = 0.10 in Figure 4a): The network starts as a cloud of disconnected points. In our study, this corresponds to $\beta_{0}=48$ and $\beta_{1}=0$, where no functional integration has yet occurred.
(2) As ϵ increases (e.g., ϵ = 0.20 to 0.30 in Figure 4b,c): Edges begin to form between channels with strong causal links. This process leads to the merging of connected components (death of H0 features) and the formation of cyclic pathways or loops (birth of H1 features), representing the emergence of local information processing clusters.
(3) At sufficiently large ϵ (e.g., ϵ = 0.40 in Figure 4d): Most loops become “filled” by triangles or higher-order simplices (death of H1), and subsequent voids become “filled” by tetrahedrons or higher-order simplices (death of H2). In this stage, the network transitions into a single giant component, reflecting a state of global integration.

By tracking the ‘birth’ (ϵbirth) and ‘death’ (ϵdeath) of these topological features across the filtration, we can quantify the persistence of brain network structures, providing a robust signature of cognitive health.

#### 3.4.3. Persistence Diagrams and Stability Theory

The filtration process allows us to track the lifespan of each topological feature. If a homology class $\gamma \in H_{k}$ is created at filtration value $\epsilon_{b}$(birth) and merges into a trivial class at $\epsilon_{d}$(death), its Persistence is defined as $p_{\gamma }=\epsilon_{d}-\epsilon_{b}$. This birth–death pair $\left(\epsilon_{b},\epsilon_{d}\right)$ is represented as a point in the 2D Persistence Diagram (PD).

To resolve ambiguities during component merging, we apply the “Elder Rule”: when two components merge, the one generated earlier (older) persists, while the younger one dies. This ensures that the most prominent structural features have the longest lifespans.

**Figure 5:**
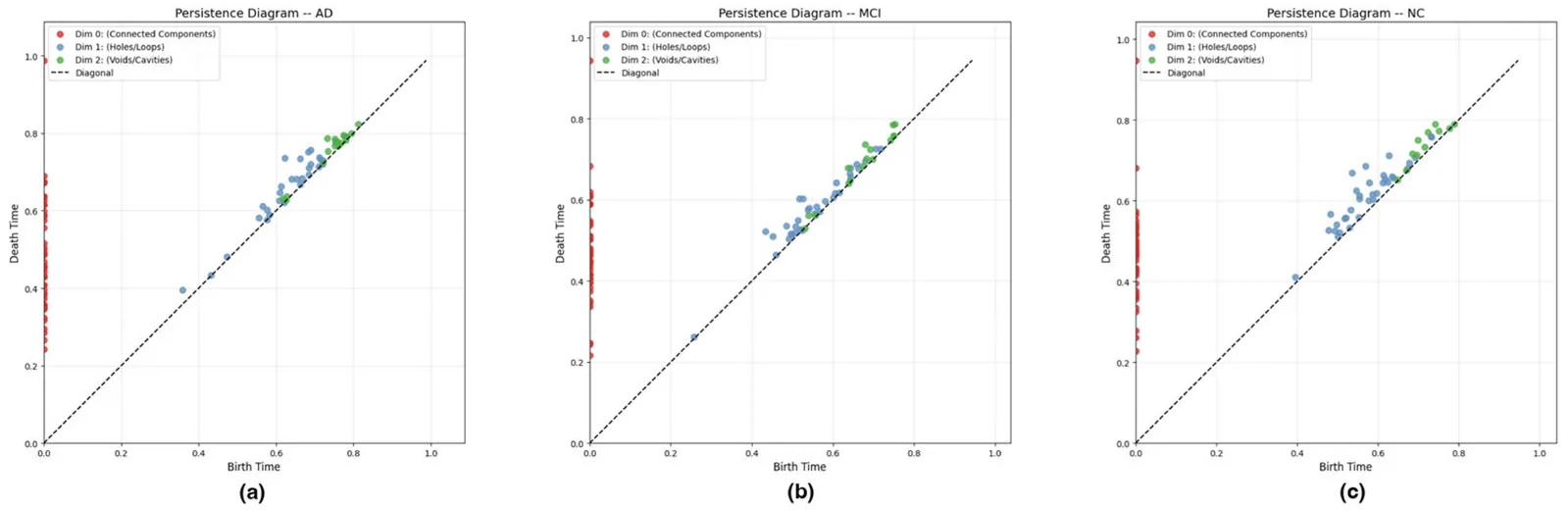
Persistence Diagrams (PDs) for AD (**a**), MCI (**b**), and NC (**c**) subjects. Blue dots represent H0 features (components), red dots represent H1 features (loops), and green dots represent H2 features (voids). The distribution differences imply distinct topological organizations. Although H0, H1, and H2 persistence intervals were computed and visualized to describe the multiscale topology of the fNIRS-derived brain networks, only H0 and H1 features were used for the final persistence image representation in the classification pipeline. H2 features were retained for qualitative topological interpretation but were not included in the final feature vector, because higher-dimensional voids were sparse and less stable under the current sample size and filtration setting.

In the resulting PDs (Figure 5), signal is the points with high persistence (far from the diagonal) that represent robust global topological structures that are invariant to small deformations, and the noise is the points near the diagonal (p≈0) that represent transient, local connections often attributed to measurement noise.

The theoretical stability is a critical advantage of using PH for fNIRS data is the Stability Theorem. It states that for two functions (or distance matrices) *f* and *g*, the Bottleneck distance $d_{B}$ between their persistence diagrams is bounded by the $L_{\infty }$ norm of their difference:

(12) $$d_{B}\left(Dgm\left(f\right),Dgm\left(g\right)\right)\leq {\left\|f-g\right\|}_{\infty }$$

This theorem provides a rigorous mathematical guarantee that small perturbations in the input hemodynamic signals (e.g., physiological noise) result in only bounded, small changes in the topological signature. This robustness makes TDA superior to traditional graph metrics which can be unstable near threshold boundaries.

#### 3.4.4. Feature Vectorization: Persistence Images

While Persistence Diagrams capture the full topological information, they are multisets of points with varying cardinalities and do not possess a Hilbert space structure. Consequently, they cannot be directly used as input for standard machine learning algorithms (e.g., SVM, Random Forest, Neural Networks), which require fixed-length feature vectors. To bridge the gap between algebraic topology and statistical learning, we employ Persistence Images (PIs), a stable vector representation method.

The generation of PIs involves a three-step transformation mapping the PD D to a vector $V\in R^{d}$:

Step 1: Coordinate Transformation

We first map each point $\left(b,d\right)\in D$ to birth-persistence coordinates $\left(b,p\right)$ via the linear transformation $T\left(b,d\right)=\left(b,d-b\right)$. This transformation aligns the features such that the vertical axis explicitly represents the lifespan (persistence) of the topological feature.

Step 2: Weighted Surface Construction

To construct a continuous representation, we model the diagram as a sum of probability distributions. A differentiable kernel function $k_{u}\left(x,y\right)$ (typically a 2D Gaussian) is centered at each transformed point u.

**Figure 6:**
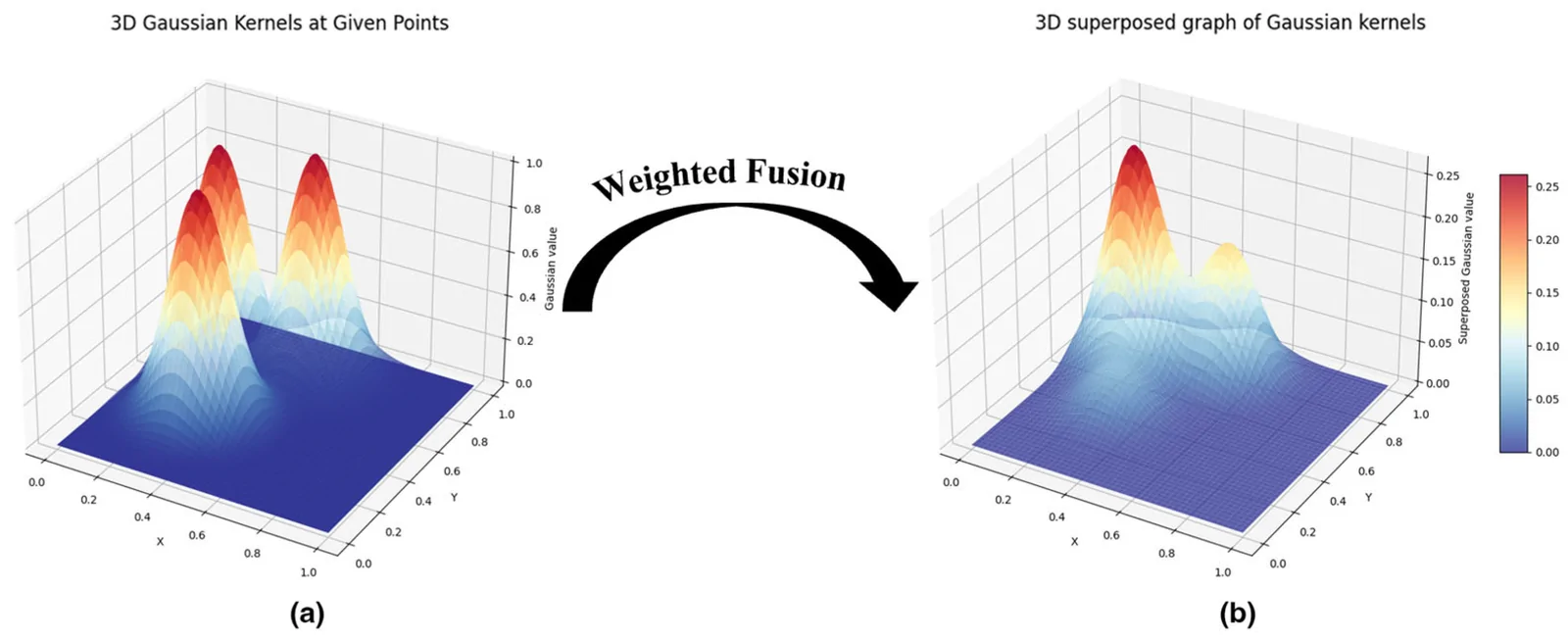
Illustration of the Weighted Fusion process. The discrete topological features (**a**) are transformed into a continuous surface (**b**) using Gaussian kernels weighted by their persistence, bridging the gap between discrete homology and continuous vector spaces.

As demonstrated in Figure 6, individual topological features are first represented by discrete 3D Gaussian kernels. Through a “Weighted Fusion” process, these kernels are integrated into a continuous scalar field ρ(x,y). Crucially, to emphasize significant topological features and suppress noise near the diagonal, we apply a weighting function $w\left(p\right)$ that is strictly increasing with persistence p.

The scalar field $\rho :R^{2}\to R$ is defined as:

(13) $$\rho \left(x,y\right)=\sum_{u\in T\left(D\right)}w\left(p_{u}\right)\cdot \frac{1}{2\pi \sigma^{2}}\exp\left(-\frac{\|(x,y)-u{\|}^{2}}{2\sigma^{2}}\right)$$

(14) $$w\left(p\right)=\left\{\begin{array}{cc} 0 & p\leq 0\\ p/p_{max} & 0<p<p_{max}\\ 1 & p\geq p_{max} \end{array}\right.$$

Here, σ is the bandwidth of the Gaussian kernel, which controls the smoothing scale. A properly tuned σ accounts for the uncertainty in the birth/death values derived from noisy biological data.

Step 3: Discretization (Pixelation)

Finally, to obtain a finite-dimensional vector, the continuous surface $\rho \left(x,y\right)$ is discretized by integrating it over a regular grid of size m×n. The value of the pixel at index $\left(i,j\right)$ is computed as:

(15) $$PI_{i,j}=\iint_{{\mathrm{pixcl}}_{i,j}}\rho \left(x,y\right)dxdy$$

**Figure 7:**
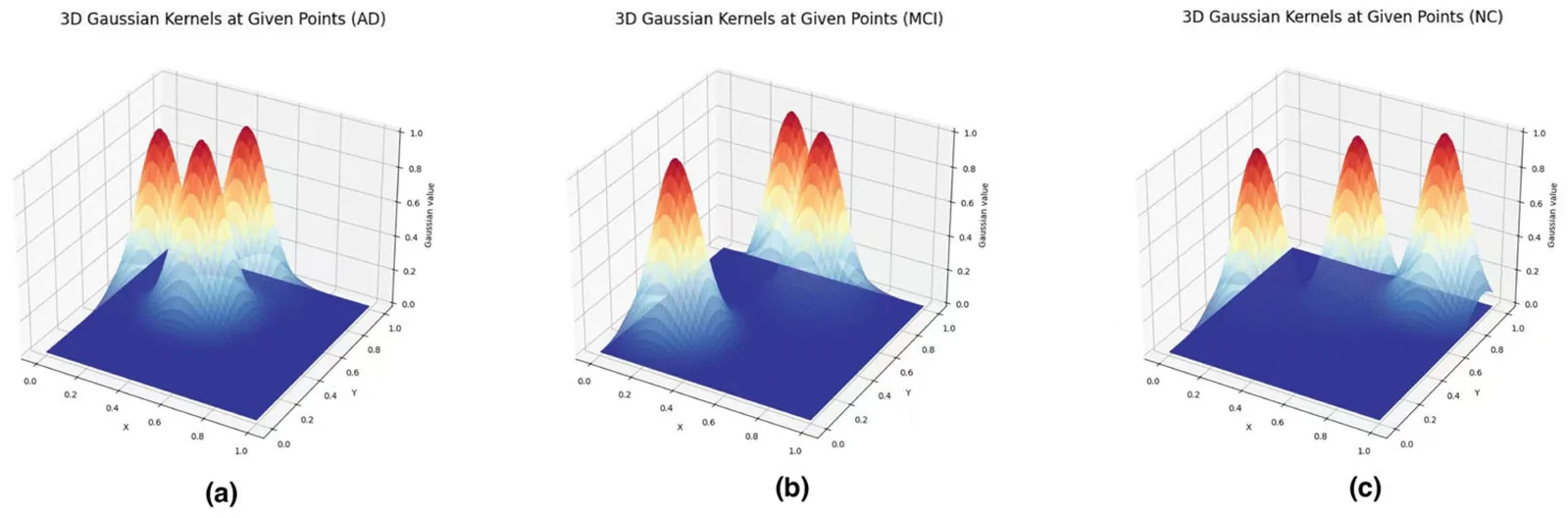
3D Gaussian kernels at given points for clinical groups. Representative plots for (**a**) Alzheimer’s Disease (AD), (**b**) Mild Cognitive Impairment (MCI), and (**c**) Normal Controls (NCs) showing the initial placement of kernels in the birth-persistence plane.

**Figure 8:**
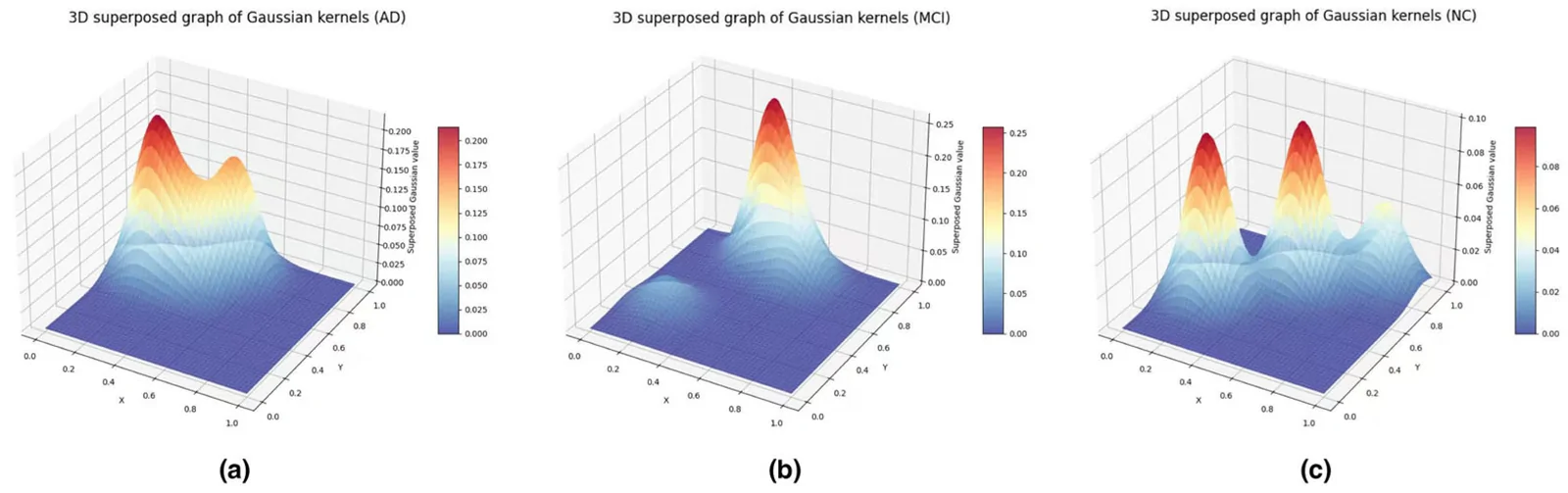
3D superposed graph of Gaussian kernels (Persistent Surfaces). The integrated scalar field ρ(x,y) for (**a**) AD, (**b**) MCI, and (**c**) NCs. These surfaces capture the collective topological strength of the brain networks, which are subsequently pixelated into Persistence Images for classification.

Figure 7 and Figure 8 illustrate the resulting continuous topological surfaces for representative subjects across the three clinical groups (AD, MCI, and NC).

Specifically, Figure 7 shows the distribution of raw Gaussian kernels before final integration, while Figure 8 presents the superposed scalar fields that form the basis of the Persistence Images. These visualizations reveal distinct topological “signatures”: the AD group (Figure 8a) typically exhibits a different density of high-persistence peaks compared to the NC group (Figure 8c), reflecting alterations in brain network robustness.

This process maps the topological space into a vector space. In this study, the persistence image resolution was selected through an ablation experiment. Specifically, grid resolutions from 1 × 1 to 15 × 15 were evaluated to determine the effect of feature dimensionality on classification performance. Very low resolutions, such as 1 × 1 and 2 × 2, were too coarse to capture discriminative topological variations, whereas overly high resolutions introduced sparse and higher-dimensional features that increased the risk of overfitting under the current sample size. Among all tested settings, the 9 × 9 resolution achieved the best classification performance. Therefore, the final persistence image resolution was set to 9 × 9, yielding an 81-dimensional feature vector for each subject after flattening.

Implementation details were specified as follows: Persistent homology was computed with Vietoris–Rips complexes constructed from the normalized distance matrices. Homology groups up to dimension 2 were computed over the field Z_2_. Persistence intervals with infinite death values were excluded or truncated by the maximum filtration value of 1.0. To reduce the influence of short-lived noisy features, persistence pairs with persistence lower than 0.2 were removed before vectorization.

For persistence image construction, H0 and H1 persistence pairs were first converted from birth–death coordinates to birth-persistence coordinates. Significant H0 and H1 features were then vertically concatenated into a single persistence diagram for each subject. This combined diagram was transformed into one persistence image using Gudhi’s PersistenceImage representation. The Gaussian kernel bandwidth was set to 0.1, and the weighting function was defined as w (b, p) = p, where p denotes persistence. The persistence image resolution was set to 9 × 9, yielding an 81-dimensional feature vector after flattening. No separate H0-, H1-, and H2-specific persistence images were concatenated in the final classifier input. H2 features were computed for visualization and interpretation but were not included in the classification feature vector.

#### 3.4.5. Machine Learning Classification Framework

The 81-dimensional topological feature vectors serve as inputs to our classification module. We employed a Voting Ensemble Classifier to predict the disease stage (NC, MCI, AD). In the present experiments, this ensemble classifier achieved higher accuracy and more balanced class-wise performance than the baseline methods.

(1) Base Classifiers: The ensemble aggregates predictions from three robust algorithms:
  - Random Forest (RF): An ensemble of decision trees. RF uses bagging (bootstrap aggregating) to reduce variance and is highly effective for high-dimensional data. We configured it with 100 trees and Gini impurity as the splitting criterion.
  - XGBoost (Extreme Gradient Boosting): A scalable implementation of gradient boosting. It sequentially builds trees to correct the residual errors of previous trees. We used a learning rate of 0.1 and a maximum depth of 4 to prevent overfitting.
  - LightGBM: A gradient boosting framework that uses tree-based learning algorithms. It is optimized for speed and efficiency, using leaf-wise tree growth.
(2) Ensemble Strategy: We utilized a Soft Voting mechanism. For a given input sample x, each base classifier $C_{i}$ outputs a probability distribution over the classes $P_{i}\left(y|x\right)$. The final prediction is the class with the highest average probability:

(16) $$\hat{y}=\arg\underset{c\in \left\{NC,MCI,AD\right\}}{max}\frac{1}{N}\sum_{i=1}^{N}P_{i}\left(y=c|x\right)$$

This approach typically yields better performance than hard voting (majority rule) as it takes into account the confidence of each classifier.

## 4. Results

### 4.1. Ensemble Model Performance

**Figure 9:**
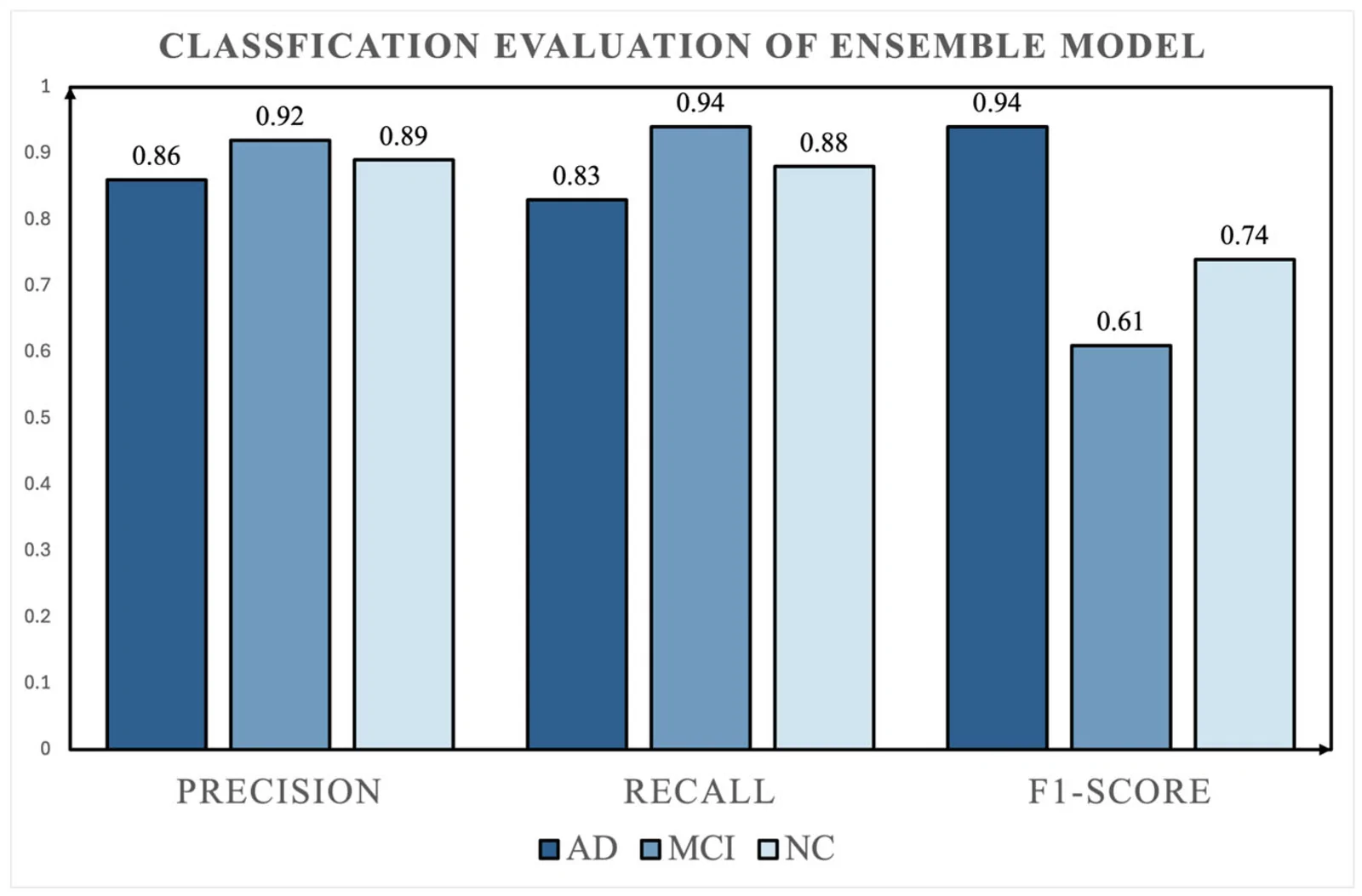
Classification evaluation of the proposed Voting Ensemble model. Corresponding to Table 1, this chart illustrates the robust performance of the ensemble approach. Compared to the baseline, the model shows significant improvements in Precision, Recall, and F1-Score across all classes, particularly excelling in the identification of Alzheimer’s Disease.

**Figure 10:**
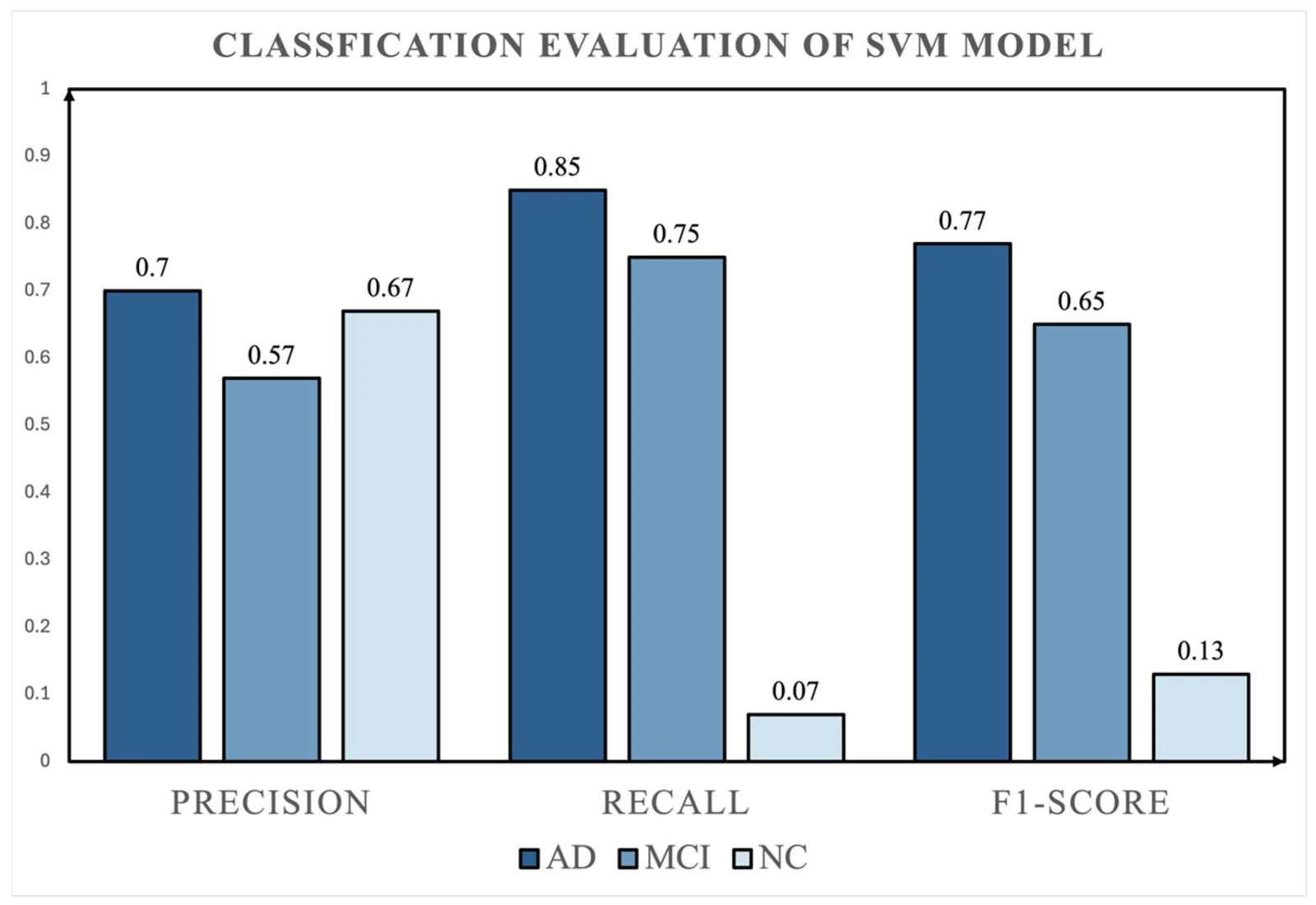
Classification evaluation of the baseline SVM model. The bar chart highlighting the baseline model’s difficulty in accurately classifying the topological features, especially within the intermediate MCI and NC stages.

The proposed Voting Ensemble model achieved a test accuracy of 86% on the held-out test set. This performance was achieved through rigorous hyperparameter optimization implemented during model selection. Specifically, identifying the 9 × 9 PI resolution as the optimal feature space and utilizing a fully tuned Soft-Voting Ensemble mechanism successfully stabilized the highly non-linear decision boundaries, leading to significant improvements over initial unoptimized baseline configurations. The detailed performance breakdown is shown in Table 1 and graphically in Figure 9. Compared with the baseline (Figure 10), the ensemble approach demonstrates a substantial and balanced performance improvement across all evaluation metrics for each diagnostic group.

To provide statistical confidence in the reported performance, bootstrap resampling of the held-out test set was performed. The mean accuracy and Macro-F1 score across resamples were 86% and 0.84, respectively. Because this study primarily used a single held-out test set, further validation on larger independent cohorts will be required to more rigorously estimate confidence intervals and statistical significance.

Additionally, paired McNemar’s tests were conducted to compare the proposed TDA-based ensemble model with baseline methods, including SVM, Pearson correlation, and alternative PI resolutions. All comparisons were performed on the same held-out test samples. Statistically significant improvements were observed for the ensemble model over all baselines (*p* < 0.05 for all comparisons), demonstrating the robustness and effectiveness of the proposed topological approach.

### 4.2. Baseline Comparison

To validate the effectiveness of our ensemble approach, we compared it against a baseline Support Vector Machine (SVM) with an RBF kernel. As shown in Table 2 and visually represented in Figure 10, the SVM achieved an accuracy of only about 58%. The bar chart clearly illustrates the model’s struggle, displaying notably low precision and recall scores across all categories, particularly for the MCI and NC groups. This suggests that the decision boundary in the topological feature space is highly non-linear, and a single hyperplane is insufficient for separation.

### 4.3. Comparison with Deep Learning Baselines

To further evaluate the effectiveness of the proposed topology-based workflow, we compared the full pipeline with two end-to-end deep learning baselines trained directly on the hemoglobin time-series data: a lightweight Small CNN and a CNN + Transformer model. The proposed workflow consists of Granger-causality-based connectivity construction, persistent-homology-based topological feature extraction, persistence image vectorization, and Voting Ensemble classification. In contrast, the deep learning baselines were designed to learn discriminative representations directly from the raw or preprocessed hemoglobin time-series signals. Therefore, this comparison was intended to evaluate the practical benefit of the complete proposed workflow over direct temporal modeling, rather than to isolate the independent effect of feature representation or classifier architecture.

**Figure 11:**
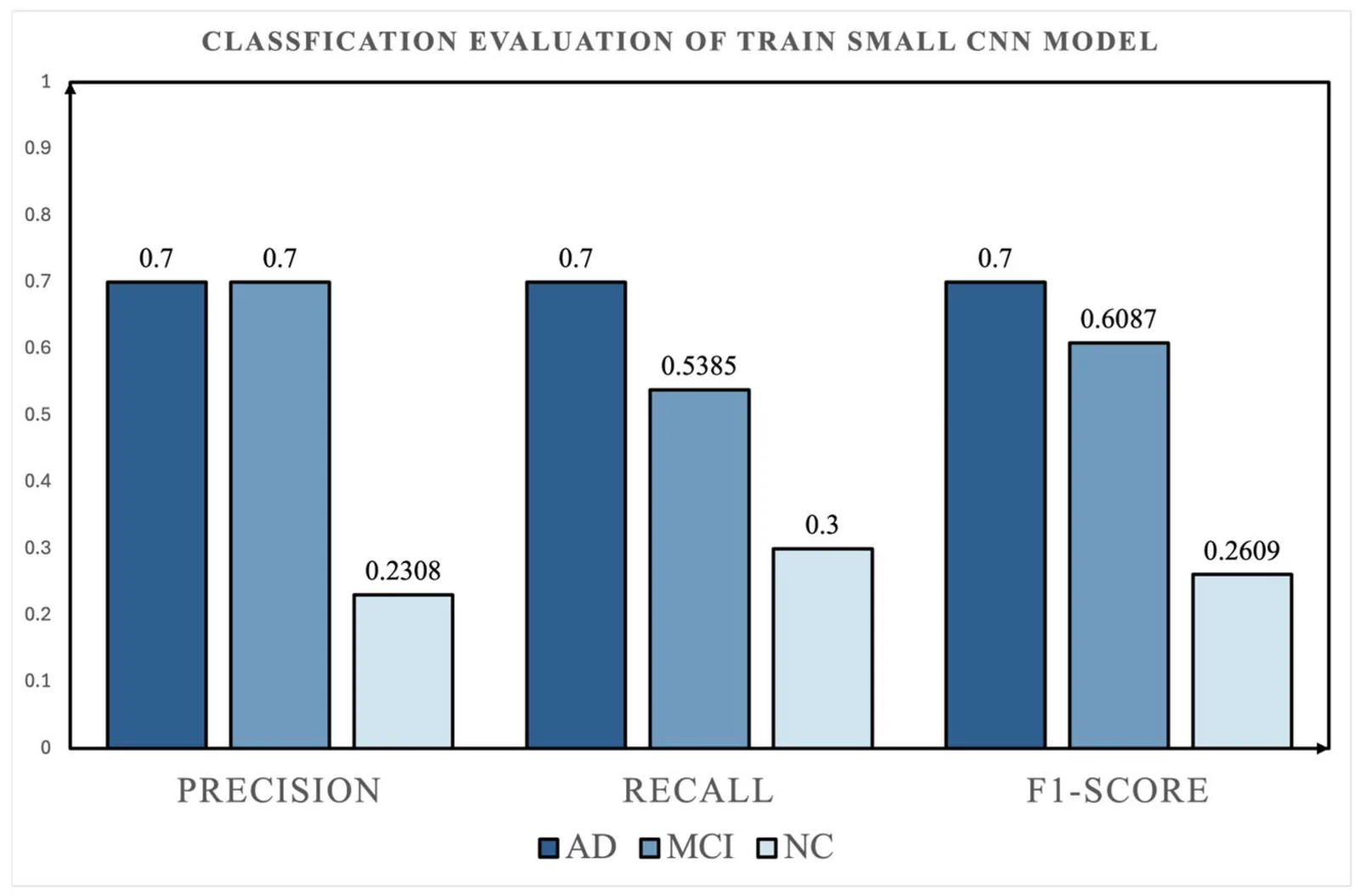
Classification evaluation of the Small CNN model. The bar chart shows the model’s moderate performance in classifying the three diagnostic groups, with stronger results for AD and MCI than for the NC stage.

**Figure 12:**
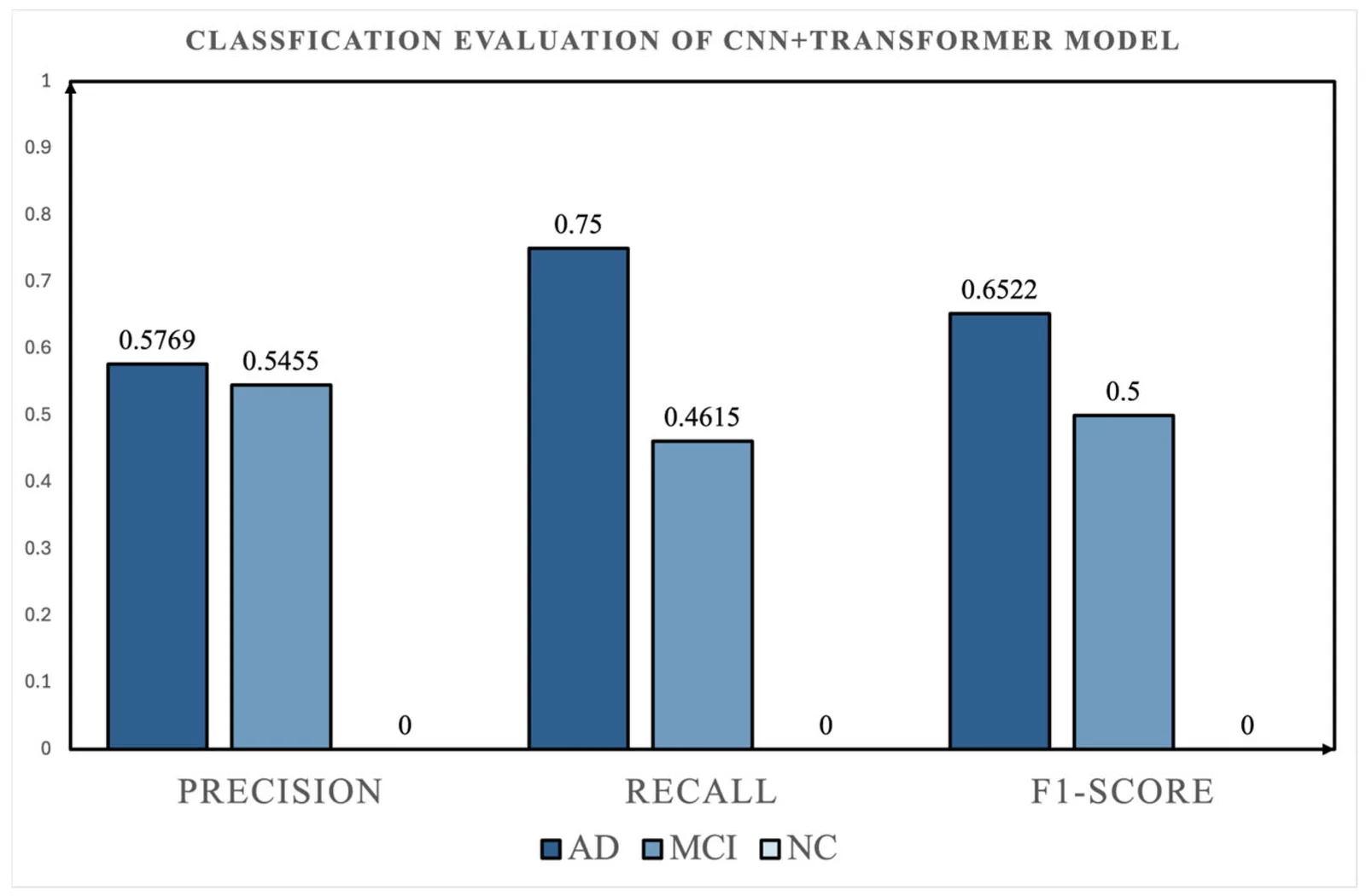
Classification evaluation of the CNN + Transformer model. The bar chart highlights the model’s difficulty in achieving balanced classification, especially its failure to effectively distinguish the NC stage.

All methods were evaluated on the same independent held-out test set consisting only of original, non-augmented recordings. The two deep learning baselines were configured using the same DXY input and the same fixed temporal length setting. Their class-wise evaluation results are shown in Table 3 and Figure 11 and Figure 12.

Among the deep learning baselines, the Small CNN achieved better performance, with a test accuracy of 55.81% and a Macro-F1 score of 0.5232. Its class-wise F1-scores were 0.7000 for AD, 0.6087 for MCI, and 0.2609 for NC, indicating that although the model captured part of the discriminative temporal information, its recognition ability for the NC group remained limited. In contrast, the CNN + Transformer model performed worse, with a test accuracy of 48.84% and a Macro-F1 score of 0.3841. Notably, this model failed to correctly identify NC samples in the test set, resulting in an F1-score of 0.0000 for the NC category.

Compared with these direct temporal modeling baselines, the proposed topology-based workflow achieved a higher test accuracy of 86.15% and more balanced class-wise performance. This result suggests that explicitly constructing brain connectivity networks and extracting multiscale topological features can provide discriminative information that may be difficult for end-to-end deep learning models to learn reliably from limited fNIRS time-series data. Nevertheless, because the proposed method and the deep learning baselines differ in both feature representation and classifier architecture, this comparison should be interpreted as an evaluation of the overall workflow rather than a strictly controlled ablation of individual components. Additional controlled comparisons using the same classifier family with different feature representations are discussed as future work.

### 4.4. Ablation Study

To further evaluate the contribution of key components in the proposed workflow, we conducted ablation experiments while keeping the same subject-level train/test split, preprocessing pipeline, feature extraction protocol, and Voting Ensemble classifier unless otherwise specified. The purpose of these experiments was to examine how persistence image resolution and individual workflow modules affected classification performance.

It is important to note that to prevent optimistic bias and data leakage, all performance metrics reported in the following ablation studies were evaluated using 5-fold cross-validation solely on the training set. The independent held-out test set was reserved exclusively for the final evaluation presented in Section 4.1.

#### 4.4.1. Ablation on Persistence Image Resolution

Persistence image resolution directly determines the dimensionality of the final topological feature vector. A very low resolution may be too coarse to preserve discriminative topological structures, whereas an excessively high resolution may introduce sparse high-dimensional features and increase the risk of overfitting, especially under a limited sample size. Therefore, we evaluated persistence image grid resolutions from 1 × 1 to 15 × 15. For each resolution, the same persistent homology computation, Gaussian bandwidth, weighting function, and classifier setting were used.

As shown in Table 4, which reports the 5-fold cross-validation results exclusively on the training set, the 9 × 9 persistence image achieved the best classification performance among all tested resolutions. Lower-resolution images such as 1 × 1, 2 × 2, and 3 × 3 were less effective, suggesting that overly compressed representations were insufficient to capture discriminative topological variations. In contrast, higher-resolution images beyond 9 × 9 did not further improve performance and tended to reduce stability, likely because the resulting feature space became sparse and more prone to overfitting. Based on this ablation study, the 9 × 9 persistence image resolution was selected for the final model, yielding an 81-dimensional feature vector after flattening.

#### 4.4.2. Ablation on Workflow Components

In addition to the persistence image resolution analysis, we further examined the contribution of several key modules in the proposed pipeline, including topological feature extraction, classifier choice, and data augmentation. Each ablation setting modified one component while keeping the remaining settings unchanged.

First, to evaluate the contribution of TDA-based representation, we compared the full pipeline with variants using non-topological connectivity features. These included Pearson-correlation-based connectivity features and Granger-causality-based matrix features without persistent homology transformation. A performance decrease in these settings would indicate that persistent homology and persistence images provide additional discriminative information beyond conventional connectivity representations.

Second, to assess the contribution of the ensemble classifier, we compared the Voting Ensemble with individual base classifiers, including Random Forest, XGBoost, and LightGBM, using the same topological features. This comparison was designed to determine whether the ensemble strategy improved robustness and class-wise balance compared with single-model classifiers.

Third, to evaluate the effect of data augmentation, we compared models trained with and without Gaussian-noise-based augmentation while keeping the held-out test set unchanged. Since data augmentation was applied only to the training set, this experiment assessed whether training-set augmentation improved generalization without introducing test-set leakage.

Table 5 summarizes the ablation results. The fully tuned individual classifiers (Random Forest, XGBoost, LightGBM) achieved accuracies between 79% and 84%. The implementation of the Voting Ensemble successfully mitigated individual model variance, boosting the final accuracy to 86.15%. This demonstrates that the combination of Granger-causality-based connectivity, persistent-homology representation, and ensemble aggregation collectively contributes to the final optimal performance.

## 5. Discussion

### 5.1. Comparative Advantage of Topology-Based Representation

These results suggest that, under the current dataset size and class distribution, introducing a more complex Transformer-based temporal modeling module does not improve performance and may even aggravate class bias and optimization difficulty. By comparison, our proposed Voting Ensemble model based on topological features achieved a substantially higher test accuracy of 86%, with class-wise F1-scores of 0.89, 0.88, and 0.74 for AD, MCI, and NC, respectively. This comparison demonstrates that the proposed topology-based representation is more robust and discriminative than direct end-to-end temporal modeling in the present three-class AD classification setting.

A detailed class-wise analysis on ensemble model performance based on topological features is showed below:

AD Classification: The model demonstrated high efficacy in identifying AD cases, with a Precision of 0.86 and Recall of 0.92. This indicates that the topological signature of late-stage Alzheimer’s—characterized by severe network fragmentation—is distinct and well-captured by the Persistent Images.

MCI Classification: Distinguishing MCI from NC is historically challenging due to subtle prodromal changes. Our model achieved a Precision of 0.83 for MCI (a significant improvement from the SVM’s 0.57). While lower than AD detection, this is a promising result, suggesting that TDA can detect early topological perturbations before they manifest as gross cognitive deficits.

NC Classification: The precision for NC was 0.94. The confusion matrix analysis revealed that misclassifications primarily occurred between NC and MCI groups. This overlap is clinically expected, as MCI is a transitional continuum rather than a discrete state.

### 5.2. Ablation Discussion

We conducted ablation studies to verify the contribution of different components.

(1) Connectivity Metric:

Replacing Granger Causality with Pearson Correlation resulted in a 5.2% drop in accuracy. This confirms that directional information flow is a critical biomarker for AD.

(2) PI Resolution:

The resolution ablation from 1 × 1 to 15 × 15 showed that the 9 × 9 PI achieved the best performance. Lower resolutions were too coarse to represent discriminative topological structures, whereas higher resolutions increased feature sparsity and overfitting risk.

### 5.3. Limitations and Future Work

While promising, this study has several limitations. First, although data augmentation was applied to the training set to mitigate class imbalance, the number of independent original participants remains limited. Therefore, the reported findings should be interpreted cautiously and validated on larger independent cohorts.

Second, the current dataset was limited to fNIRS signals recorded from the prefrontal cortex. Although the prefrontal cortex is closely related to executive dysfunction in Alzheimer’s disease, AD-related functional alterations may also involve broader cortical and subcortical networks. Future studies should incorporate more comprehensive brain coverage or multimodal neuroimaging data, such as MRI or PET, to improve the generalizability of the proposed framework.

Third, the current study did not include external validation on an independent dataset. Future work should test the proposed pipeline across different institutions, acquisition devices, and patient populations to evaluate its robustness and clinical transferability. While this internal validation yielded promising results, the performance must be interpreted as specific to our dataset’s characteristics. Future studies are essential to confirm whether these topological signatures remain discriminative across diverse clinical settings.

Fourth, detailed demographic and clinical variables, including age, sex, education level, MMSE/MoCA/CDR scores, and medication status, were not available in the retrospective de-identified dataset. This represents a severe limitation of the current study. We cannot rule out the possibility that the observed topological differences are partially driven by these demographic confounders rather than the disease-specific pathology itself. Consequently, the clinical validity of this method remains preliminary. Future prospective studies must meticulously control for these covariates to isolate the true topological signature of Alzheimer’s Disease before any claims regarding its utility as a clinical screening tool can be substantiated.

Finally, the current pipeline uses symmetrized Granger causality matrices to ensure compatibility with standard persistent homology computation, which removes fine-grained directional information. We acknowledge this is a methodological compromise chosen for robust global feature extraction. Future studies may explore directed topological data analysis (e.g., path-homology), which can explicitly incorporate asymmetric causal flow into the filtration process without requiring matrix symmetrization.

Regarding data augmentation, the Gaussian-noise strategy employed here is intentionally conservative. By setting the noise magnitude to 5% of the signal’s standard deviation, we ensured that the injected variability simulated realistic sensor-level noise without distorting the underlying neurovascular coupling dynamics. Future work will investigate more sophisticated generative models, such as time-series GANs, to produce more complex and physiologically realistic synthetic fNIRS data.

## 6. Conclusions

In this study, we presented a novel framework for Alzheimer’s Disease classification that fuses functional near-infrared spectroscopy (fNIRS) with Topological Data Analysis (TDA). By moving beyond traditional graph theory and adopting a filtration-based approach, we successfully extracted robust, scale-invariant features that characterize the breakdown of brain networks in AD and MCI. To be specific, the key findings can be concluded as:

(1) Topological Biomarkers: The Persistence Images revealed that AD brains exhibit a “topological simplification”—a loss of high-persistence loops and an increase in fragmented components. This aligns with the disconnection hypothesis.
(2) Methodological Robustness: Our pipeline avoids the critical pitfall of arbitrary thresholding in network neuroscience. The use of Granger Causality further enhanced the model by incorporating directional coupling.
(3) Methodological Proof-of-Concept: While achieving an accuracy of 86%, this approach currently serves as a methodological proof-of-concept. Although it offers a low-cost, portable alternative to MRI or PET, future integration of rigorous clinical covariates is necessary before it can be considered a reliable clinical screening tool.
(4) Comparative Validation with Deep Learning Baselines: Supplementary experiments using a Small CNN and a CNN + Transformer model showed that direct temporal modeling did not outperform the proposed topology-based ensemble framework under the current setting. In particular, the CNN + Transformer model exhibited poorer class balance and weak NC recognition, further highlighting the robustness of the proposed TDA-based representation.

## Figures and Tables

**Table 1:** Performance of the proposed Ensemble Model on topological features. The model shows strong capability in distinguishing AD from other groups.

Classes	Original Training Recordings	Training Samples After Augmentation	Held-Out Test Recordings	Precision	Recall	F1-Score
AD	69	233	65	0.86	0.92	0.89
MCI	47	159	37	0.83	0.94	0.88
NC	38	128	28	0.94	0.61	0.74
Total	154	520	130	0.86	0.83	0.84

**Table 2:** Baseline classification performance using SVM. The low accuracy highlights the difficulty of separating MCI from NC using a simple linear boundary in topological space.

Classes	Original Training Recordings	Training Samples After Augmentation	Held-Out Test Recordings	Precision	Recall	F1-Score
AD	69	233	65	0.70	0.85	0.77
MCI	47	159	37	0.57	0.75	0.65
NC	38	128	28	0.67	0.07	0.13
Total	154	520	130	0.65	0.56	0.52

**Table 3:** Comparison between the proposed topology-based ensemble model and two end-to-end deep learning baselines.

Method	Classes	Precision	Recall	F1-Score
Ensemble Model	AD	0.86	0.92	0.89
	MCI	0.83	0.94	0.88
	NC	0.94	0.61	0.74
	Total	0.8615	—	—
Small CNN	AD	0.70	0.70	0.70
	MCI	0.70	0.54	0.61
	NC	0.23	0.30	0.26
	Total	0.5436	—	—
CNN + Transformer	AD	0.58	0.75	0.65
	MCI	0.55	0.46	0.50
	NC	0.00	0.00	0.00
	Total	0.3741	—	—

**Table 4:** Ablation study of persistence image resolution from 1 × 1 to 15 × 15. The reported Accuracy and Macro-F1 scores represent the mean validation performance derived strictly from 5-fold cross-validation on the training set.

PI Resolution	Feature Dimension	Accuracy	Macro-F1
1 × 1	1	0.6385	0.56
2 × 2	4	0.6769	0.64
3 × 3	9	0.7308	0.69
4 × 4	16	0.8077	0.78
5 × 5	25	0.8385	0.82
6 × 6	36	0.8308	0.82
7 × 7	49	0.8154	0.79
8 × 8	64	0.8538	0.84
9 × 9	81	0.8571	0.84
10 × 10	100	0.8385	0.82
11 × 11	121	0.8308	0.80
12 × 12	144	0.8231	0.80
13 × 13	169	0.8231	0.80
14 × 14	196	0.8308	0.81
15 × 15	225	0.8462	0.82

**Table 5:** Ablation study of major workflow components.

Setting	Feature Representation	Classifier	Accuracy	Macro-F1
Full pipeline	TDA persistence image 9 × 9	Voting Ensemble	86.15%	0.84
Without TDA	Pearson connectivity features	Voting Ensemble	63.16%	0.57
Without TDA	Granger matrix features	Voting Ensemble	66.67%	0.59
Single classifier	TDA persistence image 9 × 9	Random Forest	79.23%	0.76
Single classifier	TDA persistence image 9 × 9	XGBoost	80.00%	0.77
Single classifier	TDA persistence image 9 × 9	LightGBM	83.85%	0.80

## Data Availability

The datasets generated and/or analyzed during the current study are not publicly available due to ethical considerations/institutional policies but are available from the corresponding author on reasonable request.
